# The millipede genus *Stemmiulus* Gervais, 1844 in Cameroon, with descriptions of three new species (Diplopoda, Stemmiulida, Stemmiulidae)

**DOI:** 10.3897/zookeys.708.14072

**Published:** 2017-10-16

**Authors:** Armand Richard Nzoko Fiemapong, Paul Serge Mbenoun Masse, Joseph Lebel Tamesse, Sergei Ilyich Golovatch, Didier VandenSpiegel

**Affiliations:** 1 Laboratory of Zoology, Faculty of Science, University of Yaounde I, P.O.Box 812, Yaounde, Cameroon; 2 Laboratory of Zoology, Higher Teacher’s College, University of Yaounde I, P.O. Box 47, Yaounde, Cameroon; 3 Institute for Problems of Ecology and Evolution, Russian Academy of Sciences, Leninsky pr. 33, Moscow 119071, Russia; 4 Royal Museum for Central Africa, Biological collection and data management unit, B-3080 Tervuren, Belgium

**Keywords:** Cameroon, key, new species, *Stemmiulus*, taxonomy

## Abstract

The large pantropical millipede genus *Stemmiulus*, which currently encompasses more than 150 species, i.e. the bulk of the species diversity of the family Stemmiulidae and entire order Stemmiulida, is shown to comprise seven species in Cameroon, including three new ones: *S. ongot* Nzoko Fiemapong & VandenSpiegel, **sp. n.**, *S. uncus* Nzoko Fiemapong & VandenSpiegel, **sp. n.**, and *S. mbalmayoensis* Nzoko Fiemapong & VandenSpiegel, **sp. n.** In addition, *S. beroni* Mauriès, 1989, previously known only from the type locality in Nigeria, is recorded from Cameroon for the first time, also being redescribed based on new samples. A key is given to all species of the genus encountered in the country, based on male gonopodal conformation, except for *S. camerunensis* (Silvestri, 1916), which was described only from female and juvenile material.

## Introduction

The Stemmiulida is a small pantropical order of Diplopoda which contains only three genera in a single family, Stemmiulidae. According to the latest classification (Enghoff et al. 2015), apart from two monobasic genera, one each in the Caribbean and Vietnam, the family is largely represented by the likewise pantropical genus *Stemmiulus* Gervais, 1844. Its 150+ species in comparable shares range from Central (one species introduced to Florida, USA) to northern South America (south to the Brazilian states of Amazônas and Bahia, as well as northern Peru), on the one hand, and Central Africa, on the other. Several *Stemmiulus* species occur in southern India and Sri Lanka, while only a few marginally also in New Guinea and the neighbouring island of Halmahera, Indonesia (Mauriès et al. 2010; Shelley and Golovatch 2011).

At present, *Stemmiulus* in Africa is comprised of 51 species or subspecies (Table 1) which range from Senegal to Tanzania and cover most of tropical Africa with the exception of southern Africa and Madagascar (Shelley and Golovatch 2011). Of them, only four species have been reported from Cameroon. The present paper puts on record three new species of *Stemmiulus* from Cameroon. In addition, *S. beroni* is found in Cameroon for the first time, also being redescribed from new samples, the first outside its type locality in Nigeria.

**Table 1.** Checklist of the African species of *Stemmiulus* with locality or country records.

1. *S. albicephalus* Mauriès, 1989; Tanzania	27. *S. mauriesi* VandenSpiegel, 2001; Kenya
2. *S. albicollis* Demange & Mauriès, 1975; Guinea and Ivory Coast (Mts Nimba)	28. *S. morbosus* (Demange & Mauriès, 1975); Guinea and Ivory Coast (Mts Nimba)
3. *S. altipratensis* (Demange & Mauriès, 1975); Guinea and Ivory Coast (Mts Nimba and Tonkoui)	29. *S. nigricollis* (Porat, 1894), sensu Mauriès (1967); Cameroon and Gabon
4. *S. aoutii* (Demange & Mauriès, 1975); Guinea and Ivory Coast (Mts Nimba)	30. *S. nimbanus* (Demange & Mauriès, 1975); Guinea and Ivory Coast (Mts Nimba)
5. *S. badonneli* (Demange & Mauriès, 1975); Guinea and Ivory Coast (Mts Nimba)	31. *S. nimbanus altipratensis* (Demange & Mauriès, 1975); Mt Nimba
6. *S. bellus* (Cook, 1895); Liberia, Mt Nimba	32. *S. oculiscaptus* Demange & Mauriès, 1975; Mt Nimba
7. *S. beroni* Mauriès, 1989; Nigeria (and Cameroon, first record)	33. *S. pencillatus* (Cook, 1895); Liberia
8. *S. calcarifer* (Demange & Mauriès, 1975); Guinea and Ivory Coast (Mts Nimba)	34. *S. perexiguus* (Demange & Mauriès, 1975); Guinea and Ivory Coast (Mts Nimba)
9. *S. camerunensis* (Silvestri, 1916); Cameroon	35. *S. perparvus* (Silvestri, 1916); Guinea
10. *S. calvus* (Cook, 1895); Liberia and Guinea (Mt Nimba)	36. *S. proximatus* (Silvestri, 1916); Cameroon
11. *S. discotarsus* VandenSpiegel, 2001; Kenya	37. *S. pullulus* (Demange & Mauriès, 1975); Guinea and Ivory Coast (Mts Nimba)
12. *S. elegans* (Silvestri, 1916); Dahomey	38. *S. ramifer* (Demange & Mauriès, 1975); Guinea and Ivory Coast (Mts Nimba)
13. *S. feae* (Silvestri, 1916); Guinea-Bissau	39. *S. recedens* (Silvestri, 1916); Guinea
14. *S. furcosus* (Demange, 1971); Sierra Leone	40. *S. regressus* (Silvestri, 1916); Guinea
15. *S. genuinus* (Silvestri, 1916); Nigeria	41. *S. royi* (Demange & Mauriès, 1975); Guinea and Ivory Coast (Mts Nimba)
16. *S. giffardi* (Silvestri, 1916) ; Ghana	42. *S. saloumensis* Mauriès, 1989; Senegal
17. *S. gilloni* (Mauriès, 1979); Senegal	43. *S. simpliciter* (Demange & Mauriès, 1975); Guinea and Ivory Coast (Mts Nimba)
18. *S. howelli* Mauriès, 1989; Tanzania	44. *S. schioetzae* (Mauriès, 1979); Sierra Leone
19. *S. infuscatus* Mauriès, 1989; Cameroon	45. *S. sjoestedti* (Brolemann, 1920); Tanzania
20. *S. jocquei* (Mauriès, 1985); Malawi	46. *S. spinogonus* Mauriès, 1989; Tanzania
21. *S. keoulentanus* (Demange & Mauriès, 1975); Guinea and Ivory Coast (Mts Nimba)	47. *S. tremblayi* (Demange & Mauriès, 1975); Guinea and Ivory Coast (Mts Nimba)
22. *S. kivuensis* Mauriès, 1989; Congo D. R.	48. *S. trilineatus* (Demange, 1971); Sierra Leone
23. *S. lacustris* (Hoffman, 1975); Rwanda	49. *S. uluguruensis* Mauriès, 1989; Tanzania
24. *S. latens* (Silvestri, 1916); Guinea-Bissau	50. *S. usambaranus* Mauriès, 1989; Tanzania
25. *S. lavellei* Mauriès, 1989; Côte d’Ivoire	51. *S. verus* Silvestri, 1916; Ghana
26. *S. lejeunei* Mauriès, 1989; Congo D. R.	

## Materials and methods

The material underlying the present contribution was collected in Cameroon in 2014–2016. All type specimens are housed in the collection of the Royal Museum for Central Africa, Tervuren, Belgium (**MBMCAS**). The samples are stored in 70% ethanol. Specimens for scanning electron microscopy (SEM) were air-dried, mounted on aluminium stubs, coated with gold, and studied using a JEOL JSM-6480LV scanning electron microscope. Photographs were taken with a Leica DFC 500 mounted on a Leica MZ16A stereomicroscope. Images were processed with Leica Application Suite. After examination, SEM material was removed from stubs and returned to alcohol, all such samples being kept in MRAC.

## Systematic account

### Order Stemmiulida Cook, 1895

#### Stemmiulidae Pocock, 1894

##### Stemmiulus

Taxon classificationAnimaliaStemmiulidaStemmiulidae

Gervais, 1844

###### Type-species.

*Iulus* (recte: *Julus*) *bioculatus* Gervais & Goudot, 1844.

###### Distribution.

Species of the genus *Stemmiulus* are know from North America (one species introduced to Florida), Central America (Mexico, Honduras, Guatemala, Costa Rica and Panama), the Caribbean (Haiti, Dominican Republic, Puerto Rico, Cuba, Guadeloupe, Virgin Islands), South America (Colombia, Ecuador, Venezuela, Guyana, Suriname, Peru and Brazil), South Asia (India and Sri Lanka), the East Indies (New Guinea and Halmahera, Indonesia), as well as tropical Africa: East Africa (Tanzania, Kenya, Malawi, Rwanda), West Africa (Nigeria, Ivory Coast, Ghana, Senegal, Sierra Leone, Guinea, Liberia, Benin?, Guinea-Bissau) and Central Africa (Congo, Gabon, Cameroon).

###### Diagnosis.

Small to medium-sized stemmiulid millipedes, reaching up 50 mm in length. Body compressed laterally, tapering gradually towards telson, metaterga striated, eyes consisting of one or two large ommatidia on each side of head.

##### Stemmiulus ongot

Taxon classificationAnimaliaStemmiulidaStemmiulidae

Nzoko Fiemapong & VandenSpiegel sp. n.

http://zoobank.org/E9E71257-DE14-426E-96B1-6F6216087D5E

Figure 1

###### Type material.

Holotype ♂ (MRAC 22734), Cameroon, Center Region, Ongot disturbed Forest, N 03°51', E 011°25', ca 810 m a.s.l., 30.I.2015, leg A. R. Nzoko Fiemapong.

Paratype: 1 ♂ (SEM, lost).

###### Etymology.

The species is named after Ongot, the type locality.

###### Diagnosis.

**Figure 1.**
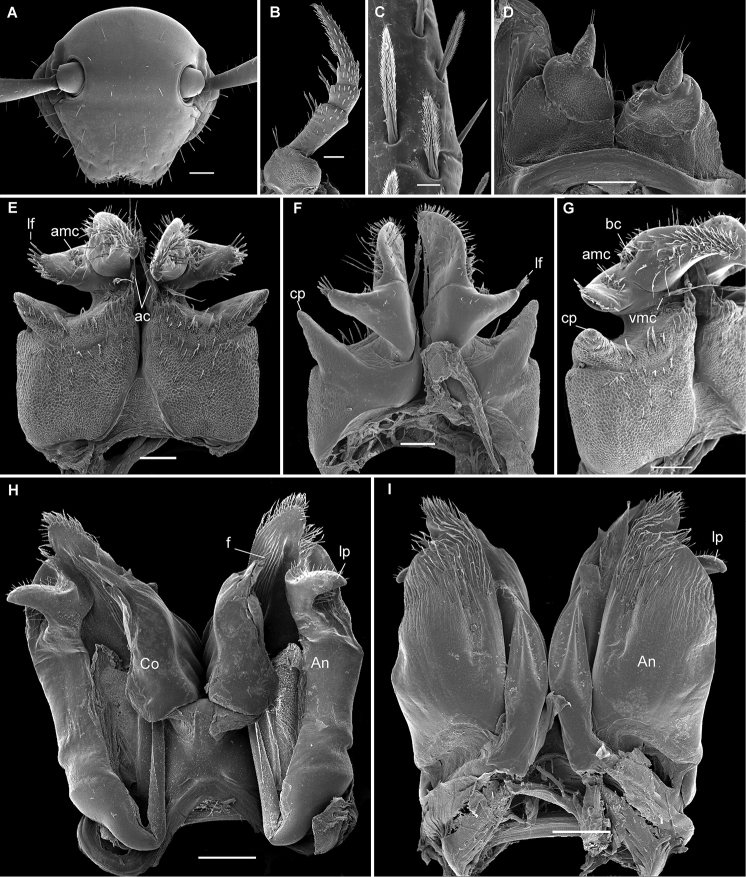
*Stemmiulus ongot* Nzoko Fiemapong & VandenSpiegel, sp. n. ♂ paratype (SEM)**. A** head front view **B** first leg-pair (one); **C** detail of the spatulate setae on the first leg-pair **D** leg-pairs 9 (paragonopods) oral view **E, F, G** leg-pair two, caudal, oral and latero-caudal views, respectively **H, I** leg-pair 8 (gonopods) caudal and oral views, respectively. Abbreviations: **ac**: apicolateral cluster of elongated setae, **An**: angiocoxite, **amc**: apicomedial cluster of setae, **bc**: basal cluster of setae, **cp**: conical projection, **Co**: colpocoxite, **f**: flagella, **lf**: lateral fringe of setae, **lp**: subterminal process, **vmc**: ventromedial cluster of setae, Scale bars 200 µm (**A, H, I**), 100 µm (**B, D–G**), 10 µm (**C**).

*Stemmiulus ongot* sp. n. is characterized by the first six pairs of male legs being densely setose, the lateral projection of the subterminal lobe of the gonopodal angiocoxites relatively short (Fig. 1H, I), the apical parts of the angiocoxite densely setose (Fig. 1H, I) and, especially, by the peculiar second pair of male legs (Fig. 1E–G), the telopodites of which are 2-segmented, the proximal segment being expanded apicolaterally and bearing a lateral fringe of setae.

###### Description.

Holotype: adult male, ca 15 mm in length, 1.7 mm in maximum diameter, body with 43 rings. Head and collum dark brown, other body rings brown with a light axial dorsal stripe, legs and antennae yellowish.

*Head* typical in shape, beset with numerous simple macrosetae (Fig. 1A); ommatidia 2+2, anterior ones slightly smaller; antennae long and setose, apices reaching fourth body ring. Gnathochilarium concave, stipes densely and uniformly porose.

*Collum* without any ornamentation. Body rings ovoid in transverse section, height/width ratio of midbody rings ca 0.41; no legless body rings in front of telson. Prozonites smooth, metazonites with oblique transverse striae.

First six pairs of *legs* covered with numerous plumose setae. First pair unmodified, tarsi with a fringe of ventral setae in basal 2/3, but forming no true brush, coxae, femora, postfemora and tibiae each with an apical cluster of prominently enlarged spatulate setae (Fig. 1B, C).

Second pair of legs with coxa enlarged and elongated, anterior face with traces of segmentation, setose over entire anterior surface, glabrous on posterior surface; laterally each produced into a prominent, elongated, conical projection (Fig. 1F, G) and with an apicomesal cluster of elongated setae. Telopodite 2-segmented, proximal segment with an apicolateral projection bearing a lateral fringe of setae, an apicomedial cluster of setae and a ventromedial cluster of long setae (Fig. 1F, G); distal segment long and slender, curved mesad, with a basal cluster of setae and plumose distally (Fig. 1G).

Pair 7 similar to following ones, without specialized setae.

*Gonopod* structure (Fig. 1H, I) typical of the genus, angiocoxite with a small, projecting, subapicolateral process. Apex of colpocoxite simple, with neither a lobe nor a projection surrounding the flagella (Fig. 1H).

*Paragonopods* small and 3-segmented, median segment carrying a short series of long setae on medial side, distal segment minute, conical, with a few apical setae (Fig. 1D).

###### Relationships.

By the relative complexity of the gonopodal structure *S. ongot* sp. n is closely related to *S. albicephalus* from Tanzania, but the striations of the lateral sides of prozonae remind of those observed in *S. infuscatus* from Cameroon. Nevertheless, the males of these species can easily be distinguished by the structure of the lateral projection of the colpocoxite which is small and apically setose in *S. ongot* sp. n., and relatively elongate without setae in *S. albicephalus* and *S. infuscatus*. On the other hand, the conformation of the second pair of legs of *S. ongot* sp. n. is unique in the entire genus *Stemmiulus*.

###### Distribution.

Known only from the type locality.

##### Stemmiulus uncus

Taxon classificationAnimaliaStemmiulidaStemmiulidae

Nzoko Fiemapong & VandenSpiegel sp. n.

http://zoobank.org/4B09E4F5-BA84-4735-B1E3-86C0E1ACE948

Figure 2

###### Type material.

Holotype ♂ (MRAC 22727), Cameroon, South Region, Vallée du Ntem Division, Engout’Adjap, N02°42', E011°09', ca 2010 m a.s.l., slightly disturbed natural forest under dead leaves, forest, 13.IX.2014, leg. A. R. Nzoko Fiemapong.

Paratypes: 1 ♂ (MRAC 22728), same data, together with holotype; 1 ♂ (SEM, MRAC 22729), same locality, but 14.III.2015, all leg. A. R. Nzoko Fiemapong.

###### Etymology.

The species name emphasizes the characteristic apical part of the colpocoxite which is unciform and pointed at the apex.

###### Diagnosis.

**Figure 2.**
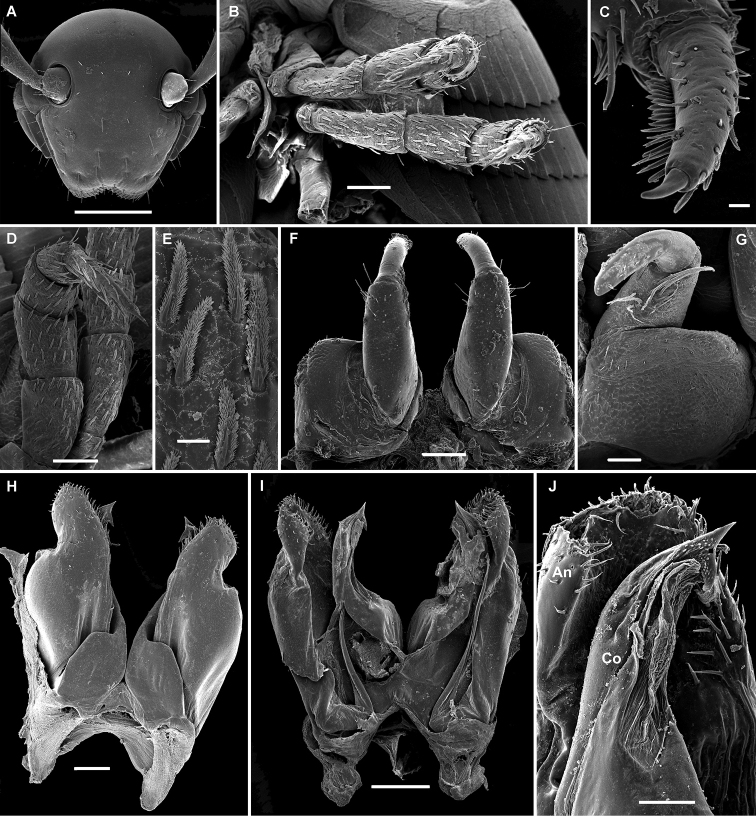
*Stemmiulus uncus* Nzoko Fiemapong & VandenSpiegel, sp. n. ♂ paratype (SEM). **A** head front view **B, D** 3 and 4 leg-pairs **C** detail of telopodite of 3 leg-pair; **E** detail of spatulate setae on the 3 leg-pair **F, G** leg-pair two, oral and caudal views, respectively **H, I** leg-pair 8 (gonopods) oral and caudal views respectively **J** apical part of right gonopod showing angiocoxite (**An**) surrounding colpocoxite (**Co**) Scale bars 500 µm (**A**), 100 µm (**B, D, F, H, I**), 50 µm (**G, J**), 20 µm (**C**).

A species of *Stemmiulus* characterized by the first six ambulatory legs being especially robust and covered with peculiar, spatulate setae, also showing a field of numerous simple setae on the inner side of the tarsus (Fig. 2B–E). The gonopod has a relatively simple angiocoxite which forms a densely setose apical corolla. The tip of the colpocoxite forms a characteristic apical hook.

###### Description.

Holotype: adult male, ca 20 mm in length, 1.8 mm in maximum diameter, body with 46 rings. Head and collum dark brown, other body rings brown with a light axial dorsal stripe, legs and antennae yellowish.

*Head* typical in shape, beset with numerous simple macrosetae; ommatidia 2+2, posterior ommatidia larger than anterior ones; antennae long and setose, apices reaching third body ring. Gnathochilarium concave, stipes densely and uniformly porose, pores surrounded by a field of minute setae.

*Collum* with a single fold at anterior edge, this being better expressed at lateral margin.

*Body* rings ovoid (height/width ratio of midbody rings ca 0.31), telson short and upcurved. Both pro- and metazonites with transverse oblique striae better pronounced at pleurotergal margin.

First six pairs of *legs* as in *S. ongot* sp. n., but mostly with filiform and plumose setae (Fig. 2B-E). First pair of legs relatively simple and unmodified.

**Figure 3.**
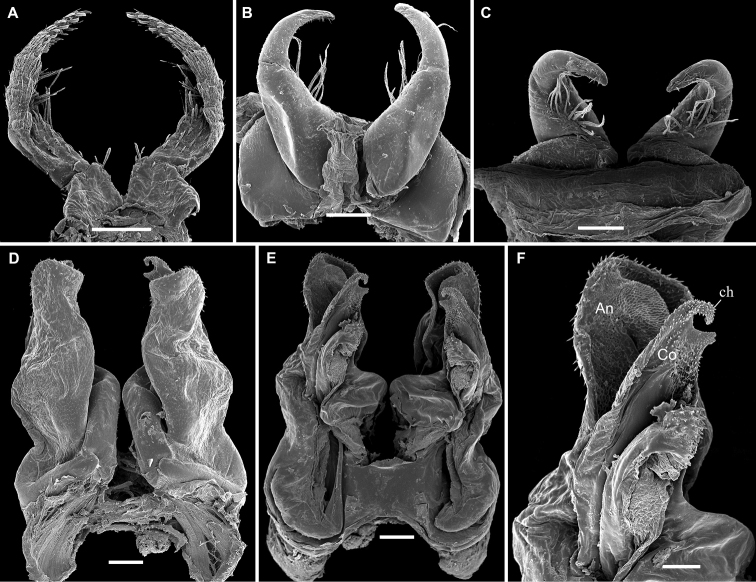
*Stemmiulus mbalmayoensis* Nzoko Fiemapong & VandenSpiegel, sp. n. ♂ paratype (SEM). **A** first leg-pair oral view **B, C** leg-pair two, oral and caudal views, respectively **D, E** leg-pair 8 (gonopods) oral and caudal views, respectively **F** apical part of right gonopod showing angiocoxite (**An**) partly surrounding colpocoxite (**Co**). Scale bars 200 µm (**A**), 100 µm (**B–E**), 50 µm (**F**).

Second pair of legs with enlarged coxae (Fig. 2F, G), their anterior surface with a few setae, posterior surface glabrous. Telopodite 2-segmented, proximal segment longer, about twice as long as distal segment, curved caudad, with a ventromedial cluster of long setae (Fig. 3G). Distal segment more slender, with an apical row of short setae (Fig. 3G).

*Gonopods* (Fig. 2H, I) with a large and relatively simple angiocoxite forming an apical corolla and covered with a dense field of numerous setae. Colpocoxite with its tip forming a characteristically strong and curved hook (Fig. 2H–J).

*Paragonopods* small and 3-segmented, each of medial and distal segments carrying a small series of short setae.

Female unknown.

###### Relationships.

The peripheral characteristics and simple gonopods bring *S. uncus* sp. n. close to *S. beroni*, from Nigeria, and *S. pullulus*, from Mount Nimba. All these species share the simplicity of their second pairs of male legs, despite the fact that the basal segment of the telopodite in the new species is about twice as large and broad as the distal segment. Nevertheless, the males of this trio can easily be distinguished by the structure of the apical part of the colpocoxite. The latter ends up in a pointed curved hook in *S. uncus*, versus a pointed straight tip in *S. beroni* or a rounded tip in *S. pullulus*.

###### Distribution.

Known only from the type locality.

##### Stemmiulus mbalmayoensis

Taxon classificationAnimaliaStemmiulidaStemmiulidae

Nzoko Fiemapong & VandenSpiegel sp. n.

http://zoobank.org/E0156B15-46D8-4349-B47C-93724027D862

Figure 3

###### Type material.

Holotype ♂ (MRAC 22730), Cameroon, Center Region Zamakoe near Mbalmayo Reserve Forest, N 03°33', E 011°31', 815 m a.s.l., forest, 19.IV.2014, leg. A. R. Nzoko Fiemapong.

Paratype: 1 ♂ (SEM, MRAC 22731), same locality, pitfall trap, 18.IV.2015, leg. A. R. Nzoko Fiemapong.

###### Etymology.

The species is named after the Mbalmayo Reserve Forest, the type locality.

###### Diagnosis.

A species close to the previous new one and to *S. beroni* by its external characters, but is easily distinguished by the structure of the colpocoxite whose apical part is axe-shaped.

###### Description.

Holotype: adult male, ca 20 mm in length, 1.8 mm in maximum diameter, body with 46 rings. Head and collum dark brown, other body rings brown with a light axial dorsal stripe, legs and antennae yellowish.

Head typical in shape, beset with numerous simple macrosetae as in previous species; ommatidia 2+2, posterior ommatidia slightly larger than anterior ones. Antennae reaching the fourth body ring, and covered with minute setae.

*Gnathochilarium* concave, without special modification, stipes densely and uniformly porose, pores surrounded by a field of setae. Collum with a single fringe at anterior edge, this being best visible laterally. Body rings ovoid (height/width ratio of midbody rings ca 0.38), metazonites with transverse oblique striae better visible at pleurotergal margin. Striations on prozonites more weakly developed than on metazonites. Annal valves beset with numerous setae.

First pair of *legs* and legs 3 to 6 as in *S. uncus* (Fig. 3A).

Second pair of legs with enlarged and subquadrate coxae (Fig. 3B, C), anterior surface with traces of segmentation, a few setae on entire anterior surface, posterior surface glabrous. Telopodite 2-segmented, proximal segment longer, about twice as long as distal one, curved ventrad, with a ventromedial cluster of long setae (Fig. 3B, C). Distal segment more slender, curved mesad, with an apical row of short setae.

*Gonopods* (Fig. 3D–F) relatively simple in structure, angiocoxite with a well prominent constriction in subapical part, apical part forming a setose corolla. Colpocoxite ending up in an axe-shaped structure slightly protruding from angiocoxite.

*Paragonopods* small, 3-segmented, quite similar to those in most of the African congeners.

Female unknown.

###### Relationships.

Most of the peripheral characters and especially the simple gonopods seem to bring *S. mbalmayoensis* sp. n. close to *S. uncus* sp. n., *S. beroni* and *S. pullulus*. Nevertheless, the males of all these species can easily be distinguished by the structure of the colpocoxite, in which the apical part is axe-shaped in *S. mbalmayoyensis* sp. n., pointed and unciform in *S. uncus* sp. n., pointed and straight in *S. beroni*, but with a rounded tip in *S. pullulus*.

###### Distribution.

Known only from the type locality.

##### Stemmiulus beroni

Taxon classificationAnimaliaStemmiulidaStemmiulidae

Mauriès, 1989

Figure 4

###### New material.

1 ♂, 1 ♀ (MRAC 22732), 1 ♂ (SEM, MRAC 22733), Cameroon, South Region, Kribi, road toward Bipindi, Bidou I, cocoa plantation, disturbed vegetation near secondary forest; N3°03'25", E10°06'02" 80 m a.s.l. collect by hand 14.X.2014, all leg. A. Henrard and VandenSpiegel.

###### Description.

**Figure 4.**
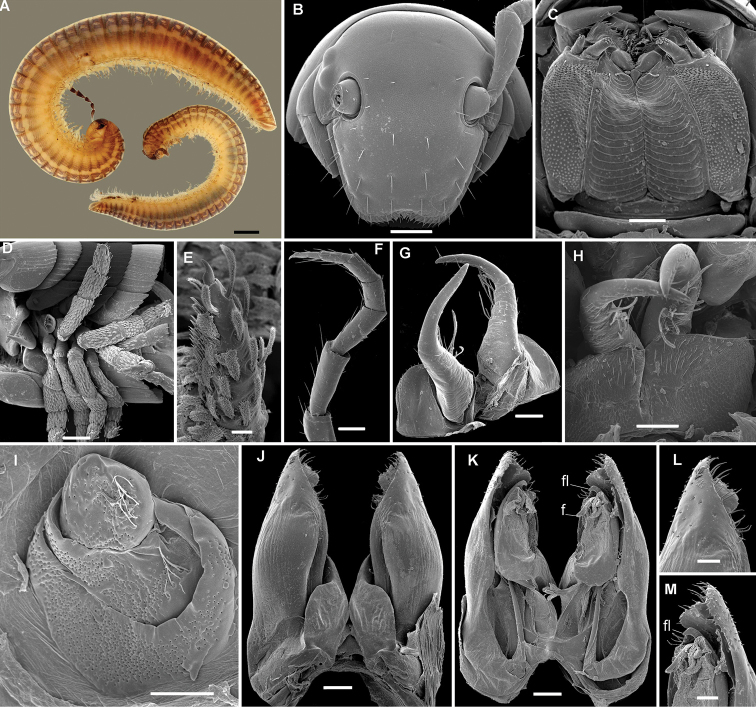
*Stemmiulus beroni* Mauriès, 1989. **A** Habituses of ♂ (small specimen) and ♀ (large specimen) **B** head front view **C** gnathochilarium **D** leg-pairs 3 to 7 **F** first leg-pair (one) **G, H** leg-pair two, oral and caudal views, respectively **I** left paragonopod, oral view **J, K** leg-pair 8 (gonopods) oral and caudal views, respectively **L, M** apical part of right gonopod oral and caudal views, respectively. Abbreviations: **f**: flagella, **fl**: finger-like process. Scale bars 1 mm (**A**), 200 µm (**B, D**), 100 µm **(C, F–H, J, K)**, 50 µm (**L, M**), 20 µm (**E**)

Adult males ca 13 mm in length, 1.5 mm in maximum diameter (height/width ratio ca 1.36), body with 43–44 rings; female with 46 rings, including 2 apodous (height/width ratio ca 1.15). Body light brown with 2–3 marbled spots lying symmetrical to mid-dorsal region which is covered by a large yellowish band all along its extent (Fig. 4A). Metazonites and dorsal margins of antennomeres darkish; legs and ventral parts of body yellowish.

*Head* typical in shape, beset with numerous simple macrosetae (Fig. 4B); ommatidia 2+2, posterior ommatidia ca 1.6 times larger than anterior ones, antennae long and densely setose. Body rings with oblique striations converging dorsad; prozonital groove weakly visible. Gnathochilarium concave, stipes densely and uniformly porose. Lingual lamellae subtrapezoidal with concave striations (Fig. 4C). Collum with a small fold at anterior edge. Body rings ovoid (height/width ratio of midbody rings ca 0.38), with transverse oblique striae better expressed at pleurotergal margin and converging anteriorly dorsad. Ozopores very small. Pygidium with 2+2 setigerous spinnerets.

First pair of male *legs* with short and globular coxae, telopoditomeres clothed with numerous plumose setae. First article of telopodite long and voluminous, nearly equal in length to all three other telopoditomeres combined, tarsal segment with a brush of setae on basal two-thirds of ventral surface.

Second pair of male legs relatively simple, with rounded coxae and 2-segmented telopodites, distal segment of the latter being relatively slender. Anterior side of proximal part of telopodite covered with long plumose setae (Fig. 4G, H).

Ventral surface of first six pairs 3 to 7 of male legs clothed with numerous plumose setae, tarsal segment with a fringe of setae in basal two-thirds of ventral surface but no true brush formed (Fig. 4D, E).

Legs 8 and following unmodified (Fig. 4F).

*Gonopods* (Fig. 4J–M) relatively simple; angiocoxite subconical, forming distally a corolla covered with a field of numerous setae. Colpocoxite shorter than angiocoxite, folded leaf-shaped, encompassing the flagellum tip and ending in a finger-like apical structure.

###### Remark.

This species is new to the fauna of Cameroon and is illustrated, based on new material taken from outside the type locality (Jos, Plateau State, Nigeria) for the first time. The fresh males from Cameroon are peculiar in the apical part of the colpocoxite being slightly curved (Fig. 4K–M), versus straight in the holotype.

### Taxonomic comments on *S. nigricollis*

Among the *Stemmiulus* species known to occur in Cameroon, *S. nigricollis* was the first to be described (Porat 1894). According to Mauriès (1967), who revised the type material of *S. nigricollis*, Porat based the description on one adult and one subadult female, both labelled “Types” and actually representing syntypes. Regrettably, there was no other geographical label given other than “Kamerun”. Working on a diplopod collection from Gabon, Mauriès (1967) discovered a species he identified as *S. nigricollis* in view of marked external similarities and the proximity of Gabon to Cameroon. He designated a male neotype from Gabon, erroneously thinking that could stabilize nomenclature. However, the act of neotype designation is only warranted when true type material is lost. Therefore, since the syntypes are still available and kept at the Stockholm Museum, the species from Gabon described by Mauriès is to be referred to as *S. nigricollis* (Porat, 1894) *sensu* Mauriès, 1967.

Since the key below is based on male characters alone, the female-based *S. camerunensis* is excluded from treatment. Silvestri (1916) described his *S. camerunensis* from a series of syntypes which included an adult female and two juveniles, all taken at Victoria, Cameroon. Only recollecting fresh topotypes, including male material, would finally allow us to clarify the identity of *S. camerunensis* and to incorporate this species into a key.

### Key to *Stemmiulus* species known to occur in Cameroon

**Table:** 

1	Angiocoxite of gonopod with a subapicolateral projection (Fig. 1H, lp)	**2**
–	Angiocoxite of gonopod without a subapicolateral projection	**4**
2	Second pair of legs relatively complex in structure, coxa with a well pronounced subconical projection anterolaterally (Fig. 1F, cp)	**3**
–	Second pair of legs relatively simple in structure, coxa without projection	***S. nigricollis***
3	Basal segment of telopodite of second pair of legs forming laterally a subconical projection with a field of localized setae on the tip (Fig. 1E–G)	***S. ongot* sp. n.**
–	Basal segment of telopodite of second pair of legs without subconical projection	***S. infuscatus***
4	Corolla of angiocoxite of gonopod with a well-pronounced constriction in subapical part (Fig. 2H, I)	***S. uncus* sp. n.**
–	Corolla of angiocoxite of gonopod without a constriction in subapical part	5
5	Apical part of colpocoxite forming a stretched finger-like process (Fig. 4K,M)	***S. beroni***
–	Apex of colpocoxite axe-shaped (Fig. 3D–F)	***S. mbalmayoensis* sp. n.**

## Supplementary Material

XML Treatment for Stemmiulus

XML Treatment for Stemmiulus ongot

XML Treatment for Stemmiulus uncus

XML Treatment for Stemmiulus mbalmayoensis

XML Treatment for Stemmiulus beroni

## References

[B1] Enghoff H Golovatch S Short M Stoev P Wesener T (2015) Diplopoda - taxonomic overview In: Minelli A (Ed.) The Myriapoda, Treatise on Zoology. Anatomy, Taxonomy, Biology. Brill, Leiden & Boston, 363–447. https://doi.org/10.1163/9789004188273_017

[B2] Mauriès JP (1967) Matériaux récoltés par M.-H. Coiffait au Gabon: Myriapoda, Diplopoda. Biologia Gabonica 3(4): 361–401.

[B3] Mauriès JP Golovatch SI Geoffroy JJ (2010) A new genus and species of the order Stemmiulida from Vietnam (Diplopoda). Arthropoda Selecta 19(2): 73–80.

[B4] Porat O (1894) Zur Myriopodenfauna Kameruns. Bihang till K. Svenska Vet. Akad. 20: 1–90.

[B5] Shelley RM Golovatch SI (2011) Atlas of Myriapod Biogeography. I. Indigenous Ordinal and Supra-Ordinal Distributions in the Diplopoda: Perspectives on Taxon Origins and Ages, and a Hypothesis on the Origin and Early Evolution of the Class. Insecta Mundi 0158: 1–134.

[B6] Sivestri F (1916) Contribuzione alla conoscenza degli Stemmiulidae (Diplopoda). Bollettino del Laboratorio di Zoologia generale agraria della R. Scuola superiore d’agricoltura in Portici 10: 287–347.

